# PEG–lipid–PLGA hybrid nanoparticles loaded with berberine–phospholipid complex to facilitate the oral delivery efficiency

**DOI:** 10.1080/10717544.2017.1321062

**Published:** 2017-05-16

**Authors:** Fei Yu, Mingtao Ao, Xiao Zheng, Nini Li, Junjie Xia, Yang Li, Donghui Li, Zhenqing Hou, Zhongquan Qi, Xiao Dong Chen

**Affiliations:** 1 Fujian Key Laboratory of Organ and Tissue Regeneration, Organ Transplantation Institute, Medical College, Xiamen University, Xiamen, China,; 2 School of Pharmaceutical Sciences, Xiamen University, Xiamen, China,; 3 Cancer Research Center, Medical College, Xiamen University, Xiamen, China,; 4 School of Basic Medical Sciences, Fujian Medical University, Fuzhou, China,; 5 Department of Biomaterials, College of Materials, and; 6 Department of Chemical and Biochemical Engineering, College of Chemistry and Chemical Engineering, Xiamen University, Xiamen, China

**Keywords:** Nanoparticles, berberine, PLGA, oral

## Abstract

The natural product berberine (BBR), present in various plants, arouses great interests because of its numerous pharmacological effects. However, the further development and application of BBR had been hampered by its poor oral bioavailability. In this work, we report on polymer–lipid hybrid nanoparticles (PEG–lipid–PLGA NPs) loaded with BBR phospholipid complex using a solvent evaporation method for enhancing the oral BBR efficiency. The advantage of this new drug delivery system is that the BBR–soybean phosphatidylcholine complex (BBR–SPC) could be used to enhance the liposolubility of BBR and improve the affinity with the biodegradable polymer to increase the drug-loading capacity and controlled/sustained release. The entrapment efficiency of the PEG–lipid–PLGA NPs/BBR–SPC was observed to approach approximately 89% which is more than 2.4 times compared with that of the PEG–lipid–PLGA NPs/BBR. To the best of our knowledge, this is the first report on using polymer material for effective encapsulation of BBR to improve its oral bioavailability. The prepared BBR delivery systems demonstrated a uniform spherical shape, a well-dispersed core-shell structure and a small particle size (149.6 ± 5.1 nm). The crystallographic and thermal analysis has indicated that the BBR dispersed in the PEG–lipid–PLGA NPs matrix is in an amorphous form. More importantly, the enhancement in the oral relative bioavailability of the PEG–lipid–PLGA NPs/BBR–SPC was ∼343% compared with that of BBR. These positive results demonstrated that PEG–lipid–PLGA NPs/BBR–SPC may have the potential for facilitating the oral drug delivery of BBR.

## Introduction

Berberine (BBR), extracted from Phellodendron Chinese and Coptis Root, has aroused wide interest due to its variety of pharmacological activities (i.e. anti-cancer (Park et al., 2012), anti-inflammatory (Wang et al., 2014), anti-diabetic (Chen et al., 2014) and anti-hyperglycemic (Dong et al., 2011)) in recent years (Kong et al., 2004). In fact, as an effective and nontoxic agent in the clinic, BBR has been extensively used to treat gastroenteritis for many years. However, similar to some other herbal products, the further development and clinical application of BBR has been limited by its poor aqueous solubility and low gastrointestinal absorption (Godugu et al., 2014).

The poor oral bioavailability of BBR can be attributed to the following aspects: (1) the first-pass effect exists both in the intestine and in the liver; (2) BBR exhibits self-aggregation, which decreases the solubility of BBR in the GI tract; (3) BBR has poor permeability across the intestinal mucous membrane and (4) BBR has been confirmed to be a P-gp substrate, which limits its transport through the gut wall (Liu et al., 2016). Apparently, developing an efficient oral BBR delivery system would be beneficial for its further development and clinical application.

Many proposed pharmaceutical preparations of BBR have also been investigated in order to enhance the oral bioavailability of BBR (Xue et al., 2013). Among these, the use of lipid-based nanoscaled delivery systems such as liposomes and nanoemulsions have shown great potential for enhancing intestinal absorption through M-cell uptake, transcellular permeation and transport through the paracellular pathway (Pund et al., 2014).

Unfortunately, these lipid-based nanoscaled drug delivery systems, incorporating lipophilic ingredients into the bilayer of phospholipids and encapsulating hydrophilic BBR into their aqueous interiors, are sensitive to be damaged by the harsh chemical and enzymatic GI environment, which would inevitably affect the effective absorption of BBR (Nguyen et al., 2014). Thus, it would be highly desirable to design effective BBR delivery systems and obtain new insights into the pharmaceutical preparation of BBR, which could avoid these disadvantages of lipid-based nanoscaled BBR delivery systems.

Unlike lipid-based nanoscaled drug delivery systems, the polymer-based NPs, consisting of biodegradable hydrophilic or hydrophobic polymers, can incorporate the pharmacologically active components with appropriate physiological stability. Despite these encouraging potential oral applications of the polymer-based NPs, encapsulating BBR into these polymeric nanoscale carriers still remains a great challenge (Nguyen et al., 2014). The common hydrophobic polymers, including poly (ɛ-caprolactone) (PCL), poly(lactide) (PLA) and poly(lactide-co-glycolide) (PLGA), are not water soluble, which make it very difficult to encapsulate hydrophilic BBR into water-insoluble polymers efficiently. Furthermore, BBR’ lack of potential functional groups for attachment to polymers makes it difficult for BBR to be encapsulated into polymeric NPs.

Recently, the drug–phospholipid complex, which was often applied to the hydrophobic modification of hydrophilic drugs, had received significant attention because of the significant improvement of drug efficacy and safety (Yu et al., 2016). Complexing BBR with phospholipids is expected to be an effective way to increase the drug affinity with hydrophobic polymetric carrier materials to improve its loading efficiency (Cui et al., 2006). Additionally, polymer–lipid hybrid drug delivery systems have been designed to take advantage of the positive attributes of both lipid-based and polymer-based carriers, which have attracted great interest for drug delivery (Hadinoto et al., 2013). All of the above encouraging reports inspired us to use the BBR– soybean phosphatidylcholine (SPC) complex as a preparation bridge between a simple drug and a sophisticated drug system by subsequently encapsulating it into polymer–lipid hybrid NPs as a synergetic platform for the effective oral delivery of BBR. Specifically, the polymer–lipid hybrid NPs are composed of (1) PLGA, a hydrophobic core, which was used to uniformly encapsulate the BBR–SPC complex; (2) SPC, an amphiphilic intermediate layer, which was used to further enhance the drug encapsulation efficiency, improve the cellular uptake (because of similar structural components between SPC and cellular membrane) and control drug release and (3) polyethylene glycol (PEG), interspersed into the lipid shell, which was used to allow the NPs to penetrate through the mucus layer rapidly. It was also expected that the polymer–lipid hybrid NPs loaded with the BBR–SPC complex (designated PLGA–lipid–PEG NPs/BBR–SPC) could effectively encapsulate the BBR–SPC complex, which would effectively protect the BBR and enhance the transportation of BBR across the epithelium.

In the present study, the BBR-complex was successfully synthesized and efficiently encapsulated into organic polymer NPs using solvent evaporation method and a notably high drug loading (∼90%) was achieved, in contrast to the low drug loading efficiency (∼37%) of BBR-loaded organic polymer NPs (PEG–lipid–PLGA NPs/BBR). To our best knowledge, this is the first report in which the BBR-complex was introduced into organic polymer NPs for the improvement of the encapsulation efficiency of BBR and controlled/sustained drug release. These hybrid NPs would act as a prospective vehicle to improve the encapsulation efficiency, gastrointestinal absorption and oral bioavailability of BBR.

## Materials and methods

### Materials

Berberine (BBR, C_20_H_18_NO_4_, MW 336) was provided by Chengdu Pufei De Biotch Co., Ltd. (Chengdu, China). Poly (D,L-lactide-co-glycolide acid) (PLGA, 100 kDa) was provided by Daigang BIO Engineer Co., Ltd. (Shandong, China). 1,2-Distearoyl-sn-glycero-3-phosphoethanolamine-*N*-methoxy(polyethyleneglycol)-2000 (DSPE-PEG) was obtained from Avanti Polar Lipids (Alabaster, AL). Soybean phosphatidylcholine (SPC, LIPOID S-100) was purchased from Lipoid GmbH (Germany). 4′,6-Diamidino-2-phenylindole (DAPI) was obtained from the Fanbo Biochemical Company (Beijing, China). Analytical HPLC-grade ethanol, formic acid, methanol and acetonitrile were purchased from Fisher Chemicals (Fair Lawn, NJ). All other chemicals and reagents were purchased from Sigma-Aldrich (St. Louis, MO) unless otherwise noted.

### Preparation of the BBR–phospholipid complex

The BBR–SPC complex was prepared by a solvent evaporation technique. First, 5 mg of BBR and 20 mg of phospholipids were dissolved in hot ethanol and dichloromethane independently. They were then mixed together into a round-bottom flask very quickly. Finally, the organic solvent was removed by rotary evaporation in a vacuum, yielding a thin film on the wall of the flask. The complex was flushed with nitrogen and then thoroughly dried to remove the residual organic solvents by placing a flask on a vacuum pump overnight. The BBR–SPC was sealed and stored at 4 °C before conducting further studies.

### Preparation of PEG–lipid–PLGA NPs/BBR–SPC

The PEG–lipid–PLGA NPs/BBR–SPC NPs were prepared *via* a solvent evaporation technique (Yu et al., 2015). First, the BBR–SPC complex was dissolved in 1 mL of ethanol and subsequently mixed with 3 mL of chloroform containing PLGA. The mixture was then added into 12 mL of a 5% ethanol aqueous solution containing SPC and DSPE–PEG at a mass ratio of 2:1, followed by probe sonication in an ice bath. The organic solvent (chloroform) in the emulsion was eliminated by evaporation with continuous stirring for 12 h at room temperature. The blank PEG–lipid–PLGA NPs were also prepared by the same procedure without adding the BBR–SPC complex.

Additionally, the hybrid NPs without the BBR–SPC complex (PEG–lipid–PLGA NPs/BBR) were prepared with the same procedure, except that the BBR–SPC was replaced by BBR. All of the above suspensions were centrifuged at 20 000 rpm for 30 min at 4 °C to collect the NPs.

### Encapsulation efficiency and drug-loading

Assays for encapsulation efficiency and drug-loading content of BBR were performed on an Agilent 1100 HPLC system. The analysis of BBR was performed with an Agilent Zorbax SB-C18 (2.1 × 50 mm, 1.8 μm) with a column temperature of 40 °C. The mobile phase was 0.1% formic acid (solvent A) and acetonitrile (solvent B). The gradient program used was as follows: 0 min, solvent A:B 75/25 (v/v); 2 min, solvent A:B 75/25; 3 min, solvent A:B 60/40; 5 min, solvent A:B 0/100; 8 min, solvent A:B 0/100; 8.01 min, solvent A:B 75/25 and 12 min, solvent A:B 75/25 (Xue et al., 2013). The flow rate was 1 mL/min. The sample injection volume was 20 μL, and detection was performed at a wavelength of 345 nm.

The percentage of BBR encapsulated in the NPs was measured directly after the complete dissolution of the NPs in dichloromethane. After evaporating off the dichloromethane solvent, the mobile phase was added to dissolve the BBR. The amount of the BBR in the mobile phase was then determined by HPLC. The entrapment efficiency was estimated by comparing the amount of BBR extracted from the NPs with the initial amount used for the NPs preparation.

### Particle size and zeta potential

The measurement of particle size and particle size distribution was assayed by the dynamic light scattering (DLS). Briefly, the NPs suspension was diluted in deionized water and then analyzed with a Zetasizer (Malvern Instruments, Malvern, Worcester-shire, UK) equipped with a 4-mW He–Ne laser operated at 633 nm through back-scattering detection. All the measurements were performed in triplicate at 25 °C.

### Scanning electron microscopy (SEM) and transmission electron microscope (TEM)

The morphology of the PEG–lipid–PLGA NPs/BBR–SPC was visualized by transmission electron microscopy (TEM, JEM 2100, JEOL, Tokyo, Japan) and scanning electron microscopy (SEM, LEO 1530VP, Elektro-nenmikroskopie GmbH, Oberkochen, Germany). The samples observed by SEM were prepared by drying the samples onto a Si substrate, followed by coating them with a 2 nm layer of Au. The images were obtained using the SEM at an acceleration voltage of 20 KeV and a secondary electron detector. The samples for TEM observation, negatively stained with 1% phosphotungstic acid, were prepared by dropping a suspension of PEG–lipid–PLGA NPs/BBR–SPC onto copper grids, which were coated with an amorphous carbon film. The sample grid was allowed to dry thoroughly at room temperature.

### Stability of PEG–lipid–PLGA NPs/BBR–SPC in simulated fluids

Stability of PEG–lipid–PLGA NPs/BBR–SPC was determined in the presence of simulated gastric fluid (SGF, pH 1.2) and simulated intestinal fluid (SIF, pH 6.8). PEG–lipid–PLGA NPs/BBR–SPC was added to simulated fluids followed by incubation at 37 °C for 2 h in SGF and 6 h in SIF. The above time intervals were selected for the study based on the expected residence time in stomach and intestine. Particle size and drug encapsulation efficiency were determined on the preset time periods.

### Differential scanning calorimetry (DSC)

Thermograms of the PEG–lipid–PLGA NPs/BBR-SPC were recorded to study the thermal behavior by a DSC 204F1 (Netzsch, Selb, Germany). PEG–lipid–PLGA NPs/BBR–SPC samples of 5–6 mg were accurately weighed and sealed in an aluminum pan. The measurements were performed with heating cycles over a temperature range of 50–400 °C at a continuous heating rate of 10 °C/min. BBR, SPC, the BBR + SPC mixture, the BBR–SPC complex, PEG–lipid–PLGA NPs and a physical mixture of all ingredients (BBR + SPC + PEG–lipid–PLGA NPs) were compared as controls (Hou et al., 2012).

### X-ray diffraction analysis (XRD)

The crystallinity and structural properties of the PEG–lipid–PLGA NPs/BBR–SPC were detected and analyzed by an X-ray diffractometer (Phillips X'pert Pro Super; Panalytical, Almelo, The Netherlands) to investigate the physical state of BBR in the PEG–lipid–PLGA NPs/BBR–SPC. The samples were scanned at the rate of 2°/min with a 0.02 step size (Ricciardi et al., 2004). BBR, SPC, the BBR + SPC mixture, the BBR–SPC complex, PEG–lipid–PLGA NPs and a physical mixture of all ingredients (BBR + SPC + PEG–lipid–PLGA NPs) were compared as controls.

### *In vitro* drug release

*In vitro* drug release was performed in SGF and SIF to simulate the physiological conditions following oral administration. The PEG–lipid–PLGA NPs/BBR–SPC were incubated at 37 °C with continuous shaking at 80 rpm with SGF or SIF in separate micro-centrifuge tubes. The PEG–lipid–PLGA NPs/BBR–SPC were separated by ultrafiltration–centrifugation using Millipore ultrafiltration centrifuge tubes (MWCO = 5000 Da). Subsequently, the samples in the ultrafiltrate were analyzed for BBR determination using the previously mentioned HPLC method (Anal & Stevens, 2005).

### Intestinal uptake study

Briefly, the formulations of BBR was given orally to SD rats at a dose of 50 mg/kg. Then, the rats were sacrificed and the selected intestinal segments were frozen in cryoembedding medium (OCT). After sectioning at 10 μm intervals (CM1900, LEICA, Germany), the sections were incubated with 4% paraformaldehyde for 10 min at room temperature and then washed three times with cold PBS (pH 7.4). Subsequently, the intestinal sections were stained with DAPI for 10 min. Finally, the samples were observed by CLSM. The channel image of berberine was performed under 488 nm laser excitation and the emission was collected within the range of 515–550 nm.

### *In vivo* pharmacokinetic studies

Male Sprague-Dawley (SD) rats (weight, 200 ± 20 g) were supplied by the Experiment Animal Center of Xiamen University. All the animal procedures complied with the guidelines of the Xiamen University Institutional Animal Care and Use Committee. Prior to drug administration, all the rats were fasted for 12 h with free access to water. The animals were kept under standard laboratory conditions (temperature of 25 ± 2 °C, relative humidity of 55 ± 5%) and were housed in polypropylene cages with free access a standard laboratory diet. The rats were divided randomly into two groups (*n* = 6). The formulations (BBR aqueous suspension, and dispersion of the PEG–lipid–PLGA NPs/BBR–SPC) were administered *via* intragastric gavage at an equivalent dose of BBR (50 mg/kg). Blood samples were withdrawn from the postorbital venous into heparinized microtubes at the following times: 0.25, 0.5, 1, 2, 4, 6, 8, 10, 12 and 24 h after dosing. The blood samples were centrifuged at 3000 rpm for 10 min at 4 °C, and the plasmas samples were transferred to clean 1.5 mL polyethylene tubes and stored at −20 °C until analysis by HPLC (Khalil et al., 2013).

### Statistical analysis

The two-tailed Student’s *t*-test was performed to analyze the statistical significances between two groups. The data are presented as the mean ± SD. A difference was considered statistically significant and highly significant when the *p* value fell below 0.05 and 0.01, respectively.

## Results and discussion

### Preparation of the PEG–lipid–PLGA NPs/BBR–SPC

Table 1 shows the effect of different weight ratio of the PLGA polymer/SPC lipid on the characteristics of the PEG–lipid–PLGA NPs/BBR–SPC with a fixed amount of PLGA. As can be observed in Table 1, not surprisingly, the isolated BBR was not easily encapsulated into the PLGA polymeric NPs. The encapsulation efficiency was only approximately 36%. This was due to the fact that it was difficult to efficiently encapsulate the hydrophilic BBR into the water-insoluble PLGA. Furthermore, the lack of potential functional groups for the attachment of BBR to the polymer macromolecules made it difficult for BBR to be encapsulated into polymeric NPs.

**Table 1.** Particle size and encapsulation efficiency of PEG–lipid–PLGA NPs/BBR–SPC at diﬀerent weight ratios of the BBR–SPC complex to PLGA polymer.

NPs	BBR–SPC:PLGA (mass ratio)	Initial drug amount (mg)	Particle size (nm)	Encapsulation efficiency (%)
PEG–lipid–PLGA NPs/BBR	–	2	173.1 ± 3.3	36.7 ± 3.4
PEG–lipid–PLGA NPs/BBR–SPC	1:8	2	161.2 ± 2.9	90.1 ± 6.2
PEG–lipid–PLGA NPs/BBR–SPC	1:4	4	149.6 ± 5.1	89.5 ± 4.6
PEG–lipid–PLGA NPs/BBR–SPC	1:2	8	151.2 ± 6.4	56.2 ± 5.9

In contrast, when the BBR–SPC complex as a bridge between free drug and drug delivery system was introduced into the PLGA polymeric NPs, the encapsulation efficiency was greatly improved. This result was likely attributed to the increased affinity and bonding forces between the BBR–SPC complex and the PLGA polymer. It was observed that the growth in the relative amount of PLGA to SPC lead to the increase in the encapsulation efficiency. Specifically, when a ratio of 4:1 or 8:1 was attained, the encapsulation efficiency was able to approach approximately 89%. In addition, the hydrodynamic particle size of the PEG–lipid–PLGA NPs/BBR–SPC was smaller than that of the PEG–lipid–PLGA NPs/BBR because of the introduction of the lipid in lipophilic kernels, which could play the role of an emulsifier.

**Figure 1.**
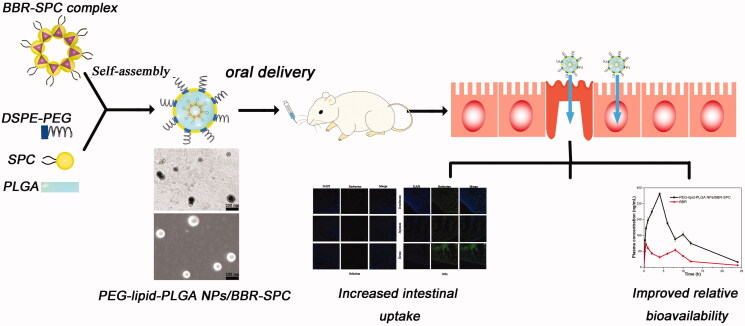
Schematic illustration of the preparation of PEG–lipid–PLGA NPs/BBR–SPC for oral drug delivery.

However, when the ratio was reduced to 2:1, the encapsulation efficiency dropped to 56.2 ± 5.9%. This result could be attributed to the fact that the relatively excess amount of the BBR–SPC complex, compared with the insufficient amount of PLGA existing in the oil phase, would tend to escape into the external aqueous phase, which might cause the self-organizing formation of phytosomes or unilamellar liposomes. In addition, the fragility of the lipid membranes of the NPs might lead to the leakage of BBR. Therefore, sufficient polymer, at least four times as much as the BBR–SPC complex, was required to entrap the BBR–SPC complex completely. Based on the results of hydrodynamic particle size and encapsulation efﬁciency, a weight ratio of PLGA/BBR–SPC complex of 4:1 was used to prepare the PEG–lipid–PLGA NPs/BBR–SPC, which were used for further studies (Figure 1).

### Characterization of PEG–lipid–PLGA NPs/BBR–SPC

**Figure 2.**
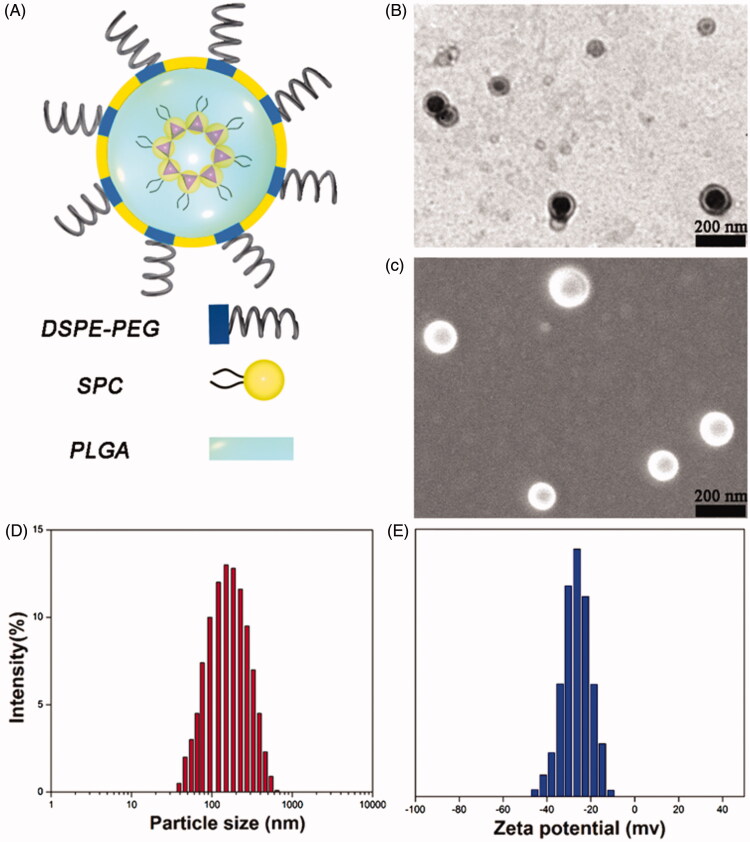
Characterization of PEG–lipid–PLGA NPs/BBR–SPC. (A) Schematic structure, (B) TEM image, (C) SEM image, (D) particle size distribution and (E) zeta potential of PEG–lipid–PLGA NPs/BBR–SPC.

The self-assembled PEG–lipid–PLGA NPs/BBR–SPC are illustrated in Figure 2(A). The sandwich-structured PEG–lipid–PLGA NPs/BBR–SPC were composed of a hydrophobic core (PLGA) loaded with the BBR–SPC complex, an amphiphilic lipid layer and a hydrophilic PEG shell. The PLGA polymer was used to encapsulate the BBR–SPC complex and provide a powerful vector to stabilize the PEG–lipid–PLGA NPs/BBR–SPC in the gastric/intestinal ﬂuids. This structure could not only control the release of BBR (drug release is discussed below), but also reduce the leakage of BBR in the preparation of PEG–lipid–PLGA NPs/BBR–SPC (encapsulation efficiency is discussed below). PEG was used to improve the transport of PEG–lipid–PLGA NPs/BBR–SPC across the intestinal epithelium (Chen et al., 2013). In addition, lipids components such as SPC/DSPE–PEG in the PEG–lipid–PLGA NPs/BBR–SPC systems, similar to the bio-membrane, were also used to increase the cellular uptake.

The mechanism of the preparation of PEG–lipid–PLGA NPs/BBR–SPC is explained below. After the introduction of ethanol into this complex, BBR–SPC was solubilized within the ethanol and subsequently developed into the reverse micelles, which were maintained even after being mixed with the PLGA oil phase. The hydrophilic head-group of SPC was directed to BBR, and the fat-soluble tail was directed toward the ethanol phase to provide the correct orientation. When the oil phase was added into the water phase, the amphiphilic lipids components, including SPC and DSPE–PEG, were adsorbed at the oil–water interface. Subsequently, their hydrophobic tails oriented to the PLGA core while the hydrophilic heads oriented to the water phase to form the PEG shell (Cheow & Hadinoto, 2011). Finally, the PEG–lipid–PLGA NPs/BBR–SPC were formed.

As shown in Figure 2(B) and (C), the optimized PEG–lipid–PLGA NPs/BBR–SPC have a uniform dispersion and a spherical shape, as determined by TEM and SEM. The mean size of the PEG–lipid–PLGA NPs/BBR–SPC was 149.6 ± 5.1 nm (Figure 2D). For oral administration, NPs with a smaller particle size could prolong the residence time in the intestinal tract and improve the intracellular uptake by intestinal epithelia (Nekkanti et al., 2016). In addition, the zeta potential of the PEG–lipid–PLGA NPs/BBR–SPC was approximately −26.8 ± 0.9 mV (Figure 2E). The measured negative potential could be caused by the carboxyl terminal of the PLGA and the negative charge on the polar head group of the phospholipid. The surface charge of NPs will also have a critical effect on the delivery of a drug across the small intestine epithelium. The interactions between positively charged NPs and the negatively charged mucus gel layer would be inevitable, which could give rise to drug release or mucosa adhesion. In contrast, NPs with a negative surface charge would easily and directly diffuse through the mucus gel layer and further diffuse to cross the intestinal epithelium. Fortunately, our PEG–lipid–PLGA NPs/BBR–SPC with negative surface charge satisfied this criterion for oral drug delivery (Shakweh et al., 2005).

The drug encapsulation efficiency is important for the drug formulation of NPs and the encapsulation efficiency is also important for further clinical applications, as more NPs would have to be consumed if the encapsulation efficiency was low. High drug entrapment efficiency would also decrease the oral dosage of the carriers and reduce side effects. When the relative amount of BBR–SPC to PLGA was 1:4, the encapsulation efficiency value was able to approach approximately 89%, which revealed that BBR–SPC played an essential role in the improvement of the encapsulation efficiency of BBR. This was likely driven by the hydrophobic interactions between the amphiphilic BBR–SPC complex and the hydrophobic segments of the polymers.

The above results indicated that our PEG–lipid–PLGA NPs/BBR–SPC had satisfied the criteria for surface charge, particle size and encapsulation efficiency for the effective oral drug delivery of BBR.

### Stability of P-BER in simulated fluids

The stability was investigated by subjecting the PEG–lipid–PLGA NPs/BBR–SPC to the simulated GIT fluids. Unlike the lipid nanoparticles, the PEG–lipid–PLGA NPs/BBR–SPC were found stable in all mediums (Table 2). The reason behind the stability could be the protective role of multiple coatings, which prevents the exposure of the BBR–SPC from the harsh conditions of the gastrointestinal tract, suggestive of the robustness of the formulation.

**Table 2.** Stability studies of PEG–lipid–PLGA NPs/BBR–SPC at SGF and SIF.

	Size (nm)		Drug encapsulation efficiency (%)	
Parameters	Initial	Final	Initial	Final
SGF pH 1.2	155.6 ± 8.10	152.6 ± 8.51	89.5 ± 4.6	76.05 ± 1.02
SIF pH 6.8	152.6 ± 4.33	145.5 ± 6.46	89.5 ± 4.6	82.91 ± 2.16

### DSC analysis

**Figure 3.**
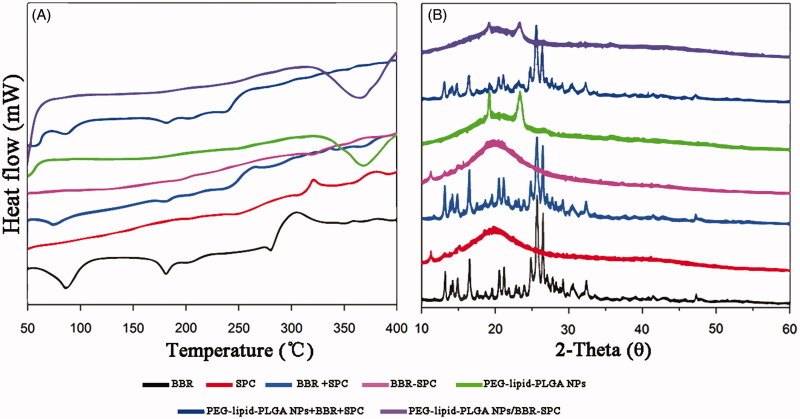
DSC and XRD of the PEG–lipid–PLGA NPs/BBR-SPC. (A) DSC and (B) XRD of BBR, SPC, BBR + SPC, BBR–SPC, PEG–lipid–PLGA NPs, a physical mixture of all ingredients and PEG–lipid–PLGA NPs/BBR–SPC.

DSC analysis was conducted to investigate the physical state of the embedded drug in the matrix of the NPs, which could influence the drug release profiles from the systems (both *in vitro* and *in vivo*). Figure 3(A) illustrates the DSC profiles of BBR, SPC, BBR + SPC, the BBR-SPC complex, PEG-lipid-PLGA NPs, a physical mixture of all ingredients (BBR + SPC + PEG–lipid–PLGA NPs) and PEG–lipid–PLGA NPs/BBR–SPC. The BBR thermal curve was typical of a crystalline substance, which can be characterized by a sharp endothermic peak (at 190 °C, which was assigned to its melting point) (Zhang et al., 2013). The strong and wide endothermic peak in the range between 90 and 120 °C could be attributed to the dehydration of BBR. In both the BBR + SPC mixture and the physical mixture of all ingredients, the melting peaks of BBR shifted slightly to a lower temperature, which could be ascribed to the weak molecular interactions between BBR and other ingredients at high temperature. In contrast, the melting peak of BBR obviously disappeared in the curve of PEG–lipid–PLGA NPs/BBR–SPC. This explained by the fact that the polymer inhibited the crystallization of BBR during the formation process of PEG–lipid–PLGA NPs/BBR–SPC. Therefore, it could be concluded that BBR in the PEG–lipid–PLGA NPs/BBR–SPC was in an amorphous or disordered crystalline phase of molecular dispersion or in a solid solution state in the polymer matrix.

### XRD analysis

To further investigate the occurrence of possible crystal structural changes, the XRD analysis was undertaken. As displayed in Figure 3(B), the free BBR showed four obvious sharp peaks, indicating the crystal nature of BBR. PEG–lipid–PLGA NPs only displayed a small and low peak at an angle of 18°, which was approximately thought to indicate an amorphous state. Some main crystalline drug signals were still detectable in both the BBR + SPC mixture and the physical mixture of all ingredients. In contrast, in the XRD diffractogram of the PEG–lipid–PLGA NPs/BBR–SPC, the crystalline peaks of BBR tended to weaken or even disappear, suggesting the conversion of the crystalline form of BBR into the amorphous form. The above result of both the DSC and XRD analysis suggested that BBR existed in the PEG–lipid–PLGA NPs/BBR–SPC matrix either in molecularly dispersed form or in an amorphous form.

### *In vitro* drug release

**Figure 4.**
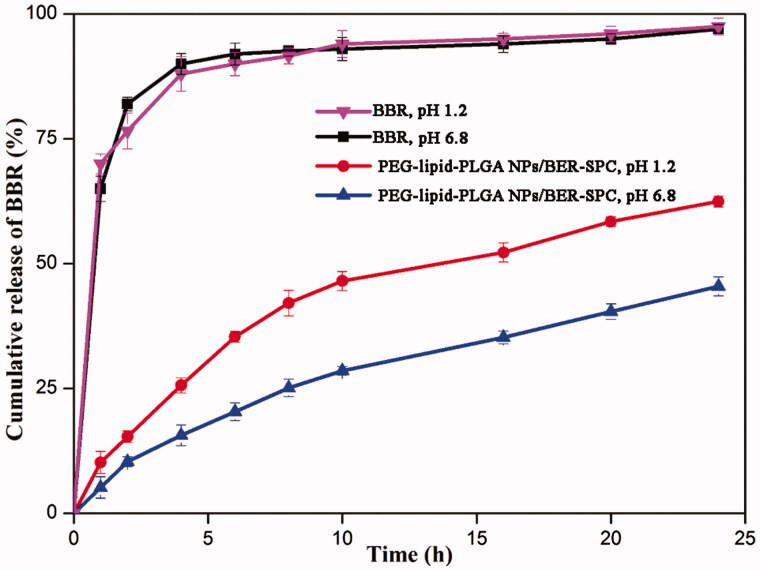
*In vitro* time-dependent drug release profiles of BBR and PEG–lipid–PLGA NPs/BBR–SPC in (A) SGF and (B) SIF.

To simulate an *in vivo* biological environment, the *in vitro* release investigations were processed in SIF (pH 6.8) and SGF (pH 1.2), respectively. The profiles of BBR released from PEG–lipid–PLGA NPs/BBR–SPC and free BBR in the two media are shown in Figure 4. Apparently, the cumulative release of BBR from the PEG–lipid–PLGA NPs/BBR–SPC was much slower than that of free BBR, which indicated that PEG–lipid–PLGA NPs/BBR–SPC could protect BBR effectively in the harsh acidic environment. The slower dug release was not only attributed to the encapsulation effect of PEG–lipid–PLGA NPs/BBR–SPC for BBR but also ascribed to the affinity between the BBR–SPC complex and PLGA. In the SGF (pH 1.2), the amount of BBR released from PEG–lipid–PLGA NPs/BBR–SPC was about 60% over 24 h. Additionally, the amount of BBR released from the PEG–lipid–PLGA NPs/BBR–SPC was approximately 40% over 24 h in the SIF (pH 6.8). The result could be ascribed to the penetration of the excess hydrogen ions, present in the SGF, into the interior of the PEG–lipid–PLGA NPs/BBR–SPC, which would contribute to the broken down of the NPs. In addition, the ester hydrolysis could be catalyzed by the acid, which would be beneficial to the de-polymerization of the PLGA polymer (Ford Versypt et al., 2013). A biphasic release pattern was also observed, which was characterized by an initial burst release of BBR at the beginning phase followed by a delayed release of BBR at the late phase (up to 24 h). The initial burst release could be attributed to the fact that some of the BBR–SPC complex was trapped on the surface of the PEG–lipid–PLGA NPs/BBR–SPC.

According to the above analysis, the delayed release of BBR in the SGF (pH 1.2) could ensure that a sufficient high concentration of PEG–lipid–PLGA NPs/BBR–SPC was absorbed by the small intestine. Therefore, the PEG–lipid–PLGA NPs/BBR–SPC had a potential ability to act as a long-acting and effective BBR delivery system *in vivo*.

### Intestinal uptake study

**Figure 5.**
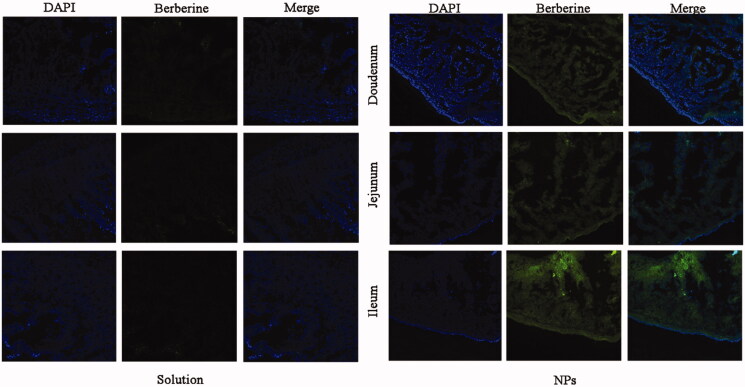
CLSM images of different segments of the small intestine. CLSM images showing the absorption of (A) BBR and (B) PEG–lipid–PLGA NPs/BBR–SPC in different segments of the small intestine. DAPI was used to label the cell nuclei (blue).

The intestinal uptake of BBR and PEG–lipid–PLGA NPs/BBR–SPC was investigated using the confocal laser scanning microscopy images of different intestinal sections at 2 h post dosing. The images in the first column display the blue fluorescence from the cell nuclei stained by DAPI. The images in the second column display the green fluorescence from BBR. The images in the third column are merged images of the first and second images. As shown in Figure 5, compared with the free BBR solution, a much stronger green fluorescence signal could be observed in the duodenum, jejunum and ileum after BBR was loaded in the PEG–lipid–PLGA NPs/BBR–SPC. This result demonstrated that the intestinal absorption of BBR was markedly increased by the PEG–lipid–PLGA NPs/BBR–SPC. The enhanced absorption of BBR might be attributed to the fact that the modified PEGylated PEG–lipid–PLGA NPs/BBR–SPC could enhance BBR’ hydrophilicity and decrease the mucoadhesion by reducing hydrophobic or electrostatic interactions (Cu & Saltzman, 2009). In addition, after oral administration of PEG–lipid–PLGA NPs/BBR–SPC, the green fluorescence was highest in the ileum, which might be due to the abundant Peyer’ patches and M-cells in the ileum (Zhang et al., 2015).

### *In vivo* pharmacokinetic study

**Figure 6.**
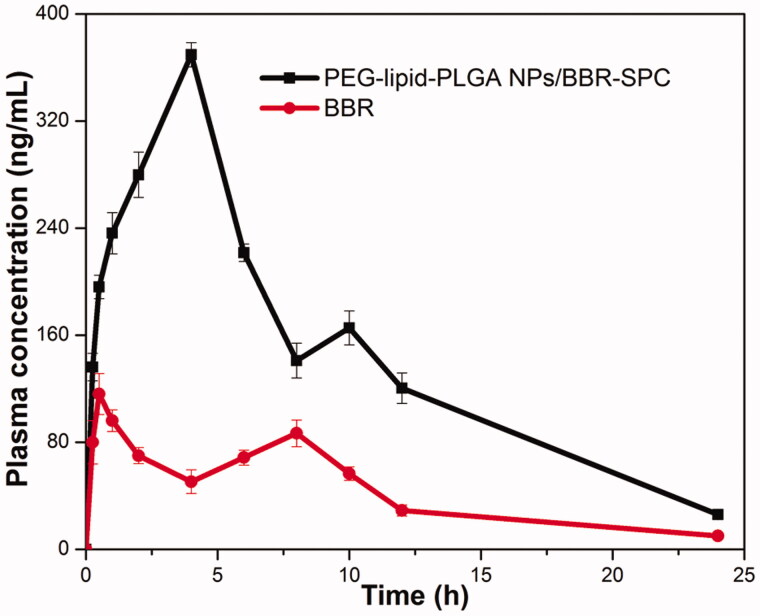
The profiles of the plasma BBR level versus time among the rats after oral administration of BBR and PEG–lipid–PLGA NPs/BBR–SPC. The data are presented as the mean ± SD (*n* = 6).

The mean plasma concentration versus time profiles of BBR after administration of the PEG–lipid–PLGA NPs/BBR–SPC and a pure drug suspension at a dose of 50 mg/kg are depicted in Figure 6. The pharmacokinetic parameters obtained from the study are summarized in Table 3. The *C*_max_ and *T*_max_ of the BBR suspension were found to be 116.09 ± 15.31 ng/mL and 0.5 h, respectively. In contrast, significantly higher *C*_max_ (369.51 ± 9.10 ng/mL) and *T*_max_ (4 h) were attained by the PEG–lipid–PLGA NPs/BBR–SPC group. Meanwhile, a higher AUC_0–_*_t_* (3499.68 ± 220.21 ng h/mL) was achieved with the PEG–lipid–PLGA NPs/BBR–SPC as compared with the BBR suspension, which only achieved 1029.03 ± 126.02 ng h/mL. In addition, an approximately 3.4-fold increase in relative bioavailability was achieved with in the PEG–lipid–PLGA NPs/BBR–SPC group when compared with the free BBR suspension group.

**Table 3.** Pharmacokinetic parameters of various groups of rats after a single oral dose administration of BBR and the PEG–lipid–PLGA NPs/BBR–SPC.

	Unit	BBR	PEG–lipid–PLGA NPs/BBR–SPC
Dose	mg/kg	100	100
*C*_max_	ng/mL	116.09 ± 15.31	369.51 ± 9.10
*T*_max_	H	0.5	2
AUC	ng h/mL	1029.03 ± 126.02	3499.68 ± 220.21
BA_R_		–	3.43 ± 0.29

AUC: area under the BBR plasma concentration–time curve; BA_R_: the relative bioavailability of the PEG–lipid–PLGA NPs/BBR–SPC compared with the oral administration of BBR group (*n*** **=** **6 for each group).

The delayed *T*_max_ with the PEG–lipid–PLGA NPs/BBR–SPC revealed the sustained drug release *in vivo*, which was in accordance with the previous *in vitro* release proﬁles. It was also attributed to the amount of time for the PEG–lipid–PLGA NPs/BBR–SPC to be taken up by M-cells before reaching the lymphatic transport system and then circulating in the blood (Sun et al., 2016). The improved *C*_max_ and AUC, obtained with the PEG–lipid–PLGA NPs/BBR–SPC, could be ascribed to: (1) the efficient absorption by virtue of their small size; (2) the modified lipophilicity and similarity between the PEG–lipid–PLGA NPs/BBR–SPC and the bio-membranes, which would facilitate adsorption and (3) the PEG–lipid–PLGA NPs/BBR–SPC overcoming the first pass effect effectively.

## Conclusions

In the current study, a novel self-assembled PEG–lipid–PLGA NPs/BBR–SPC for encapsulating the BBR–SPC complex has been developed. Small particle size, high encapsulation efficiency, good biological stability and sustained drug release characteristics have been achieved. After oral administration to rats, the oral bioavailability of the PEG–lipid–PLGA NPs/BBR–SPC was found to have been significantly enhanced, compared with that of BBR suspensions. Overall, our present work might provide a good strategy for oral delivery of BBR. The PEG–lipid–PLGA NPs/BBR–SPC may represent a nice platform for oral delivery of BBR in future.
